# 2006 Formative evaluation and adaptation of a safe sleep intervention for infants in rural underserved communities

**DOI:** 10.1017/cts.2018.76

**Published:** 2018-11-21

**Authors:** Rosemary Nabaweesi, Mary Aitken, Keneshia Bryant-Moore, Geoffrey M. Curran

**Affiliations:** 1 University of Arkansas Translational Research Institute; 2 University of Arkansas for Medical Sciences; 3 Veterans Affairs, University of Arkansas for Medical Sciences

## Abstract

OBJECTIVES/SPECIFIC AIMS: This abstract describes a recently-funded 2 year study that aims to: (1) explore the community advisors’ perspectives of the safe sleep intervention’s acceptability, feasibility, and adaptability using focus groups and key informant interviews. (2) Adapt the selected safe sleep interventions (SSI) and identify promising implementation strategies to support it through an evidence-based quality improvement process with a multistakeholder group. METHODS/STUDY POPULATION: Background sudden unexpected infant death (SUID) is the leading cause of post-neonatal infant death in the United States. Sudden infant death syndrome (SIDS), accidental suffocation and strangulation in bed account for over 50% of SUID, leading to recommendations for supine sleep position and safer sleep environments for infants. However, despite significant reductions in SIDS after “back to sleep” and “safe to sleep” campaigns, significant racial and urban-rural disparities persist. In 2015, the rural-urban crude death rate ratio was 4:1 and Black infants are twice as likely to die from SUID as White infants. Adherence to safe sleep recommendations is highly variable and a number of hospital and community-based interventions have been suggested to improve knowledge and change parent behavior. Hospital programs to promote safe sleep education and policies may serve to educate families about safe sleep, but may not be uniformly available in rural and underserved areas. The AAP evidence-based safe sleep guidelines have demonstrated reductions in SIDS and SUID when child caregivers adhere to them. Community-based SSI, including safety baby showers, promote safe sleep practices, but barriers may exist for participation, especially in rural areas. Partnering with community groups serving a high risk area, we will explore the barriers and facilitators to more widespread safety baby shower (SBS) delivery/adoption in rural underserved communities (RUC). Observation of the evidence-based SBS as it is currently delivered, focus groups and key informant interviews will be conducted with program leaders and participants. Based on this knowledge and using an evidence-based development process, we will adapt the SBS and identify implementation strategies to support its uptake in RUC. RESULTS/ANTICIPATED RESULTS: We expect to develop a modified safe sleep intervention that reaches more expectant and new mothers is more efficient at delivering safe sleep guidelines to rural community members and can be more readily adopted and implemented by RUC. Supporting implementation strategies will be identified during the formative evaluation. DISCUSSION/SIGNIFICANCE OF IMPACT: Developing a safe sleep intervention adapted for the local context through a collective decision-making process between intervention experts and local community advisors will potentially improve safe sleep guideline delivery and adherence in RUC. The next study will pilot test the effectiveness of the adapted safe sleep intervention with identified supporting implementation strategies.

